# Mechanical Loading Synergistically Increases Trabecular Bone Volume and Improves Mechanical Properties in the Mouse when BMP Signaling Is Specifically Ablated in Osteoblasts

**DOI:** 10.1371/journal.pone.0141345

**Published:** 2015-10-21

**Authors:** Ayaka Iura, Erin Gatenby McNerny, Yanshuai Zhang, Nobuhiro Kamiya, Margaret Tantillo, Michelle Lynch, David H. Kohn, Yuji Mishina

**Affiliations:** Department of Biologic & Materials Sciences, School of Dentistry, University of Michigan, Ann Arbor, Michigan, United States of America; University of Notre Dame, UNITED STATES

## Abstract

Bone homeostasis is affected by several factors, particularly mechanical loading and growth factor signaling pathways. There is overwhelming evidence to validate the importance of these signaling pathways, however, whether these signals work synergistically or independently to contribute to proper bone maintenance is poorly understood. Weight-bearing exercise increases mechanical load on the skeletal system and can improves bone quality. We previously reported that conditional knockout (cKO) of *Bmpr1a*, which encodes one of the type 1 receptors for Bone Morphogenetic Proteins (BMPs), in an osteoblast-specific manner increased trabecular bone mass by suppressing osteoclastogenesis. The cKO bones also showed increased cortical porosity, which is expected to impair bone mechanical properties. Here, we evaluated the impact of weight-bearing exercise on the cKO bone phenotype to understand interactions between mechanical loading and BMP signaling through BMPR1A. Male mice with disruption of *Bmpr1a* induced at 9 weeks of age, exercised 5 days per week on a motor-driven treadmill from 11 to 16 weeks of age. Trabecular bone volume in cKO tibia was further increased by exercise, whereas exercise did not affect the trabecular bone in the control genotype group. This finding was supported by decreased levels of osteoclasts in the cKO tibiae. The cortical porosity in the cKO bones showed a marginally significant decrease with exercise and approached normal levels. Exercise increased ductility and toughness in the cKO bones. Taken together, reduction in BMPR1A signaling may sensitize osteoblasts for mechanical loading to improve bone mechanical properties.

## Introduction

Bone mass along with bone quality is one of the determining factor of biomechanical properties and bone mineral density (BMD) has been used in clinic to predict fracture risk [1]. Mechanical loading, such as exercise, is one of the crucial factors controlling bone mass [2,3]. Reducing mechanical stress on bone leads to significant bone loss, as evidenced by osteoporosis in bedridden patients and in astronauts [4,5,6]. It is also known that Bone Morphogenetic Protein (BMP) signaling is important in regulating bone development and controlling bone mass [7,8] due to the ectopic bone forming ability of these molecules [9]. Based on their osteogenic activities [10,11], BMP2 and 7 have been used for over a decade in the clinic for bone regeneration, including applications in spine fusion and fracture healing [12].

Contrary to expectations, we found that osteoblast-specific knockout of the BMP type IA receptor, *Bmpr1a* (cKO) showed increased trabecular bone volume via decreased osteoclastogenesis [13–15], and BMP signaling was found to negatively regulate bone mass via *Sost* expression, an inhibitor for the canonical Wnt pathway. Osteoblast-specific disruption of *Bmpr1a* reduces production of RANKL, leading to the decreased osteoclastogenesis in the cKO bones [13–15]. An increase in cortical porosity was also identified in the cKO bones [13], implying that biomechanical properties may be compromised because structural integrity of the cortical compartment is necessary to bear loads [16]. It has been suggested that BMP signaling and mechanical loading cooperatively regulate downstream signaling events [17–19]. Since mechanical stimulation reduces *Sost* expression *in vivo* [20], we hypothesized that bones from *Bmpr1a* cKO mice respond to mechanical loading (exercise) to further reduce *Sost* expression, leading to increased bone mass and increased mechanical properties in the cKO bones. To test this hypothesis, we exercised cKO mice on a treadmill and examined bone structure and biomechanical properties compared to normal and non-exercised control mice.

## Materials and Methods

### Mice and exercise schedules

A transgenic mouse line expressing the tamoxifen (TM)-inducible Cre fusion protein Cre-ERTM under the control of a 3.2kb mouse procollagen a1(I) promoter (Col1-CreERTM) was bred with floxed *Bmpr1a* mice [13,14,21]. The mice had a combination of 129S6 and C57BL6/J backgrounds. They were housed in cages in a 20°C room with a 12 hour light/dark cycle.

Homozygous male mice with a floxed allele of *Bmpr1a* (*Bmpr1a* fx/fx), aged 9–10 weeks (17 *Col1-CreERTM* positive (cKO) and 14 *Col1-CreERTM* negative (control) mice) were divided randomly into two groups: exercised (Exe, n = 8 for cKO, 6 for control) and non-exercised (Nex, n = 9 for cKO, 8 for control). All mice were injected with TM (T5648, Sigma, St. Louis, MO, USA, 75 mg/kg) intraperitonially beginning at 9-weeks of age, twice a week for 2 weeks, then once a week during exercise to activate Cre recombinase activity (S1 Fig). The exercised groups of mice ran for 6 weeks, from 11 to 16-weeks of age, on a motor-driven treadmill (Columbus Instruments, Exer-6M Treadmill) for 5 days/week. Each exercise session lasted 30 minutes and the average speed was 12 ±1.0 meter/min at a 5°incline [22–24]. One week after the end of the exercise regime, at age 17 weeks, all the animals were euthanized by inhalation of carbon dioxide followed by bilateral pneumothorax, and femora and tibiae were harvested. Left tibiae were wrapped with Calcium-PBS soaked gauze and stored at -20°C until micro computed tomography (μCT) and mechanical tests were performed. Right tibiae were placed in TRIzol (Invitrogen, Grand Island, NY) and immediately crushed with a polytron for RNA extraction. Right femora were fixed with 4% paraformaldehyde and subjected to histological analyses after decalcification with 10% EDTA. All animal experiments were performed in accordance with University of Michigan guidelines covering the humane care and use of animals in research. All animal procedures used in this study were approved by University Committee on Use and Care Animals at the University of Michigan (Protocol #PRO00005716).

### MicroCT (μCT) analysis

Tibiae (n = 31, n≥6 per group) were embedded in 1% agarose, placed in a 19 mm diameter tube and scanned over the entire length of the bones using a μCT system (μCT100 Scanco Medical, Bassersdorf, Switzerland). Scan settings were: voxel size 12 μm, medium resolution, 70 kVp, 114 μA, 0.5 mm AL filter, and integration time 500 ms. A 0.6 mm region of trabecular bone was analyzed immediately below the growth plate using a fixed global threshold of 23% (230 on a grayscale of 0–1000); and a 0.4 mm region of the cortical compartment was analyzed around the midpoint of the tibia using a fixed global threshold of 30% (300 on a grayscale of 0–1000). Analysis was performed using the manufacturer’s software to obtain bone volume (BV/TV), trabecular thickness (TbTh), bone mineral density (BMD) and structural model index (SMI). Cortical geometry at the mid-diaphysis was further analyzed from thresholded slice images using a custom Matlab script. Measured properties included cortical area, cortical thickness, anterior–posterior (AP) width, medial–lateral (ML) width, bending moment of inertia about the AP and ML axes (IAP, IML), and the distance between the centroid and the anterior surface (for use in calculating material level mechanical properties). Cortical porosity was calculated by dividing the volume of the thresholded bone by the total cortical bone volume, excluding the marrow cavity, and given as a percentage (1-(BV/TV)*100).

### Histological analyses and osteoclastic analysis

Femora (n = 31, n≥6 per group) were fixed in 4% paraformaldehyde, decalcified with 10% EDTA, embedded in paraffin and sections were cut at 7 μm. These sections were stained with hematoxylin and eosin, Masson trichrome staining, or Tartrate-Resistant Acid Phosphatase (TRAP) (Leukocyte Phosphatase Staining Kit: Sigma Diagnostics). Osteoclast numbers were counted in the area 200 μm to 1200 μm from the growth plate in the distal metaphysis (n≥3 per group), and osteoclast numbers per total bone surface were measured in the same area [25] using ImageJ software.

### Quantitative real time PCR (qRT-PCR)

Whole tibiae, including marrow, were crushed by Polytron PT (Kinematica) and total RNA was extracted using TRIzol (Invitrogen) and 20% phenol. Aliquots of 1.0–1.5 μg RNA were reverse transcribed to cDNA using the SuperscriptII (Invitrogen). PCR reactions, data quantification, and analysis were performed according to the manufacturer’s standard protocol for TaqMan gene expression assays (Applied Biosystems). 40 cycles was used in PCR. Values of each mRNA were normalized to GAPDH expression in real time-based RT-PCR with the following Taqman probes: runt-related transcription factor 2 (*Runx2*); Mm00501578_m1 (115bp), osterix (*Sp7*); Mm00504574_m1 (137bp), osteocalcin (*Bglap2*); Mm01741771_g1 (77bp), metalloproteinase-9 (*Mmp9*); Mm00600163_m1 (107bp), tartrate resistant acid phosphatase (*Trap*); Mm00475698_m1 (79bp), receptor activator of NFkappaB ligand (*Rankl*); Mm00441908_m1 (69bp), osteoprotegerin (*Opg*): Mm00435452_m1 (119bp) and Mm99999915_g1 for *Gapdh*. All measurements were performed in duplicate and analyzed using the 2 ^-ΔΔCt^ method [26].

### Transmission electron microscopy and collagen fibrils analysis

Samples obtained from left femora (n≥3 per group) were subjected to transmission electron microscopy (TEM) analysis. The proximal halves of the femora were decalcified in neutral buffered 10% EDTA. The samples were postfixed with 1% osmium tetroxide in cacodylate buffer, rinsed in water, dehydrated through graded ethanol solutions, transferred to propylene oxide, and embedded in epoxy resin (EMbed 812, Electron Microscopy Sciences). Ultrathin sections were cut using a diamond knife, contrasted with uranyl acetate and lead citrate, and then examined with a CM-100 Philips electron microscope (Eindhoven, The Netherlands). Multiple micrographs of nonmineralized-bone collagen fibrils were chosen randomly and photographed at 46,000-fold magnification. Diameters of collagen fibrils were measured in the cortical compartment [27]. Mean diameter, range, and frequency distribution profiles were obtained by manually measuring the diameter of more than 500 collagen fibrils from each group.

### Mechanical testing; 4-point-bending

After μCT analyses, the left tibiae (n = 31, n≥6 per group) were mechanically tested to failure in four-point bending using displacement control (0.025mm/s) with a 3mm loading span and 9mm outer support span (Admet eXpert 450 Universal Testing Machine; Norwood, MA). Bones were aligned in the tester with the medial surface in tension and the tibia-fibula junction aligned with the outside edge of the distal loading roller. Force-displacement curves were recorded during each test and analyzed using a custom MATLAB (MathWorks, Natick, MA) script to determine whole bone strength (force), deformation (displacement), stiffness (slope of the linear region of the curve) and work (area under the curve). The yield point was defined using the 0.2% offset method [28]. The site of fracture for each bone was measured using digital calipers as the distance from the origin of fracture on the medial surface to the most distal point of the bone. This distance was used to identify the fracture location in each bone’s μCT scan. From this location, the bending moment of inertia (about the anterior-posterior axis) and distance from the centroid to the tensile (medial) surface were calculated using a custom MATLAB script and used with Euler-Bernoulli beam theory to normalize whole bone measures to tissue level properties (stress, strain, Young’s modulus and toughness) [22–24,29].

### Statistical analysis

Statistical analysis was performed using a 2-way ANOVA and Fisher’s PLSD test, and p values <0.05 were considered statistically significant. Data are presented as mean ±SEM of independent replicates (n ≥6 for histological and biomechanical analyses, n ≥3 for gene expression analyses).

## Results

### Exercise increases body weight in *Bmpr1a* cKO more than that in controls

Male *Bmpr1a* cKO (cKO, *Bmpr1a fx/fx; Col1-CreERTM(+)*) and controls (*Bmpr1a fx/fx; Col1-CreERTM(-)*) were randomly divided into two groups (Exercised and Non-exercised, Exe and Nex hereafter). There were no differences in gross morphology or body weight among these 4 groups of mice at 9 weeks of age, before TM treatment (S2 Fig). At 17-weeks of age, all mice exhibited increased body weight. Exe control and Exe cKO mice showed a significant increase in body weight in comparison to Nex control and Nex cKO (p<0.05, p<0.001, respectively) (S2 Fig). The difference in body weight between exercised and non-exercised mice was equivalent in KO and control mice.

### Exercise increases trabecular bone volume and thickness in the proximal tibia of *Bmpr1a* cKO

**Fig 1:**
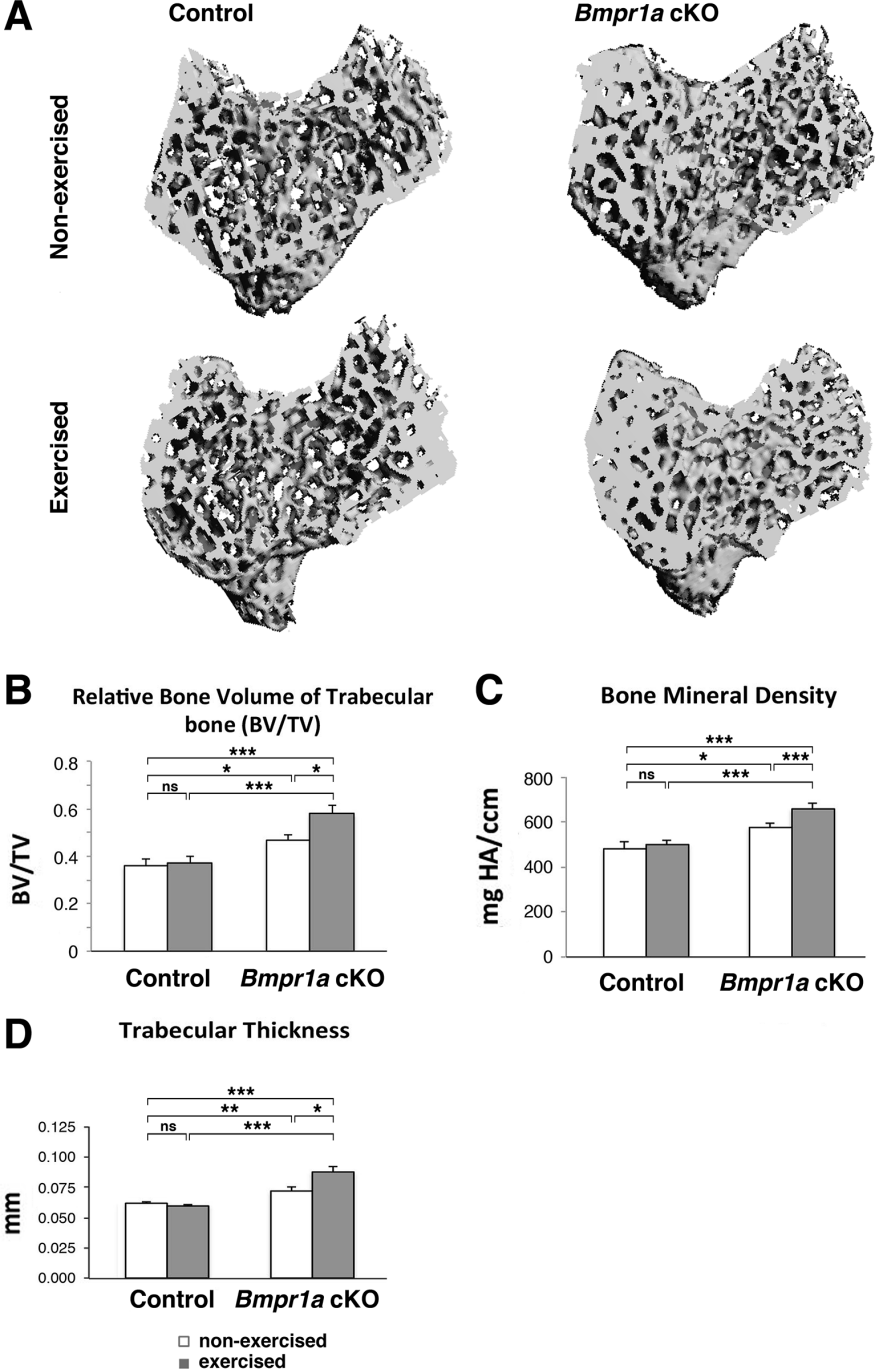
Mechanical loading increases trabecular bone volume and thickness in proximal tibial bone in *Bmpr1a* cKO bone. Trabecular architecture was measured in the following groups: *Bmpr1a* cKO mice undergoing exercise or no-exercise regimes compared to control mice also undergoing either an exercise or no exercise regime (n = 6–11 per group). (A) Representative 3D μCT images of the proximal metaphyseal regions of the tibia. (B) Measurement of bone volume per tissue volume (BV/TV). (C) Measurement of bone mineral density (BMD). (D) Measurement of trabecular thickness (TbTh). Mean±SEM, *, p<0.05, **, p<0.01, ***, p<0.001, ns, not significant.

Trabecular architecture was measured and representative 3D μCT images of the proximal metaphyseal regions of the tibia were shown (Fig 1A). In the Nex groups, the ratio of trabecular bone volume to total tissue volume (BV/TV) in the proximal tibia of the cKO mice increased by 30% (p<0.05) compared with control mice (Fig 1B). The cKO bones also showed a significant increase in trabecular bone mineral density (BMD, p<0.01) and in trabecular thickness (TbTh, p<0.05) (Fig 1C and 1D). Exercise increased BV/TV by 24% in the cKO tibiae (p<0.05), while BV/TV in the control mice was not affected by exercise (Fig 1B). Additionally, the cKO bones showed a significant increase in bone mineral density (BMD, p<0.05) and in trabecular thickness (TbTh, p<0.001) with exercise, while no changes were seen in those measurements in control bones with exercise (Fig 1C and 1D) Trabecular number (TbN) was not different among the four genotype/exercise groups (not shown). For TbTh, significant interaction by 2-way ANOVA revealed that exercise status interacts with genotype to modulate TbTh.

### Exercise decreased cortical porosity in the cKO bones towards control level

**Fig 2:**
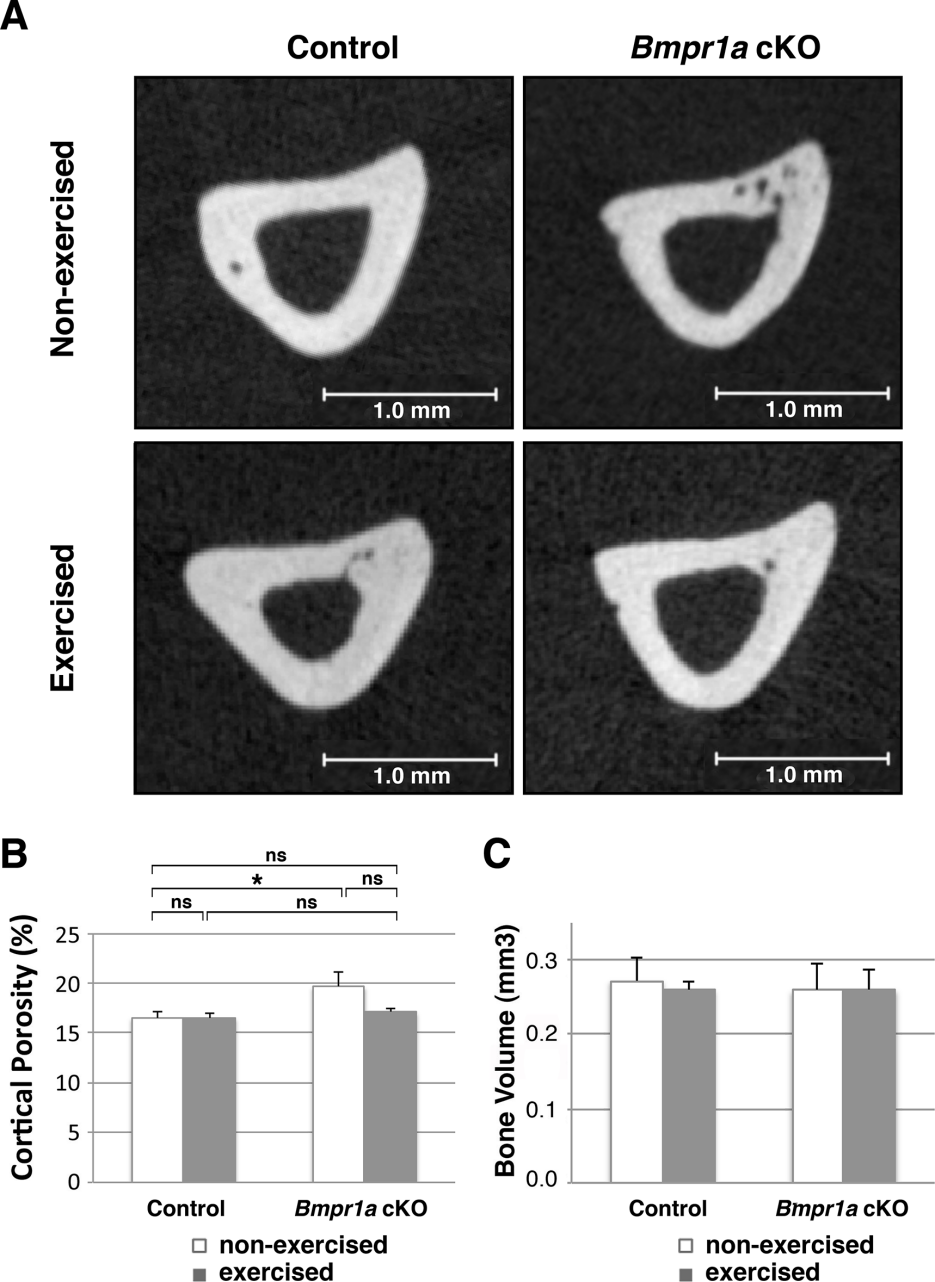
Mechanical loading decreased cortical porosity in *Bmpr1a* cKO bones. (A) Representative 2D μCT images of the cortical compartment of the tibial diaphysis. Several small pores were seen in the non-exercised cKO bone. Bones from *Bmpr1a* cKO mice undergoing exercise or no-exercise regimes were compared to bones from control mice also undergoing either an exercise or no exercise regime. (B) Measurement of cortical porosity. (C) Measurement of cortical bone volume. Mean±SEM, *, p<0.05, ns, not significant.

Next, we examined the impact of exercise on the cortical compartment of the tibial diaphysis using μCT. No differences existed in cross-sectional geometry, bone density or cortical thickness amongst the four genotype/exercise groups (Fig 2C, S3 Fig). There was a significant increase in cortical porosity in the Nex cKO tibia compared with controls (p<0.05, Fig 2A and 2B), despite no change in cortical bone volume (Fig 2C). Exercise did not alter cortical bone volume in either cKO or control mice (Fig 2C), but the porosity in the cKO bones showed a marginally significant decrease with exercise, and thus a trend to adapt to normal levels with exercise (p = 0.061) (Fig 2A and 2B).

### Exercised *Bmpr1a* cKO mice showed a lower osteoclast number than control mice when exercised

**Fig 3:**
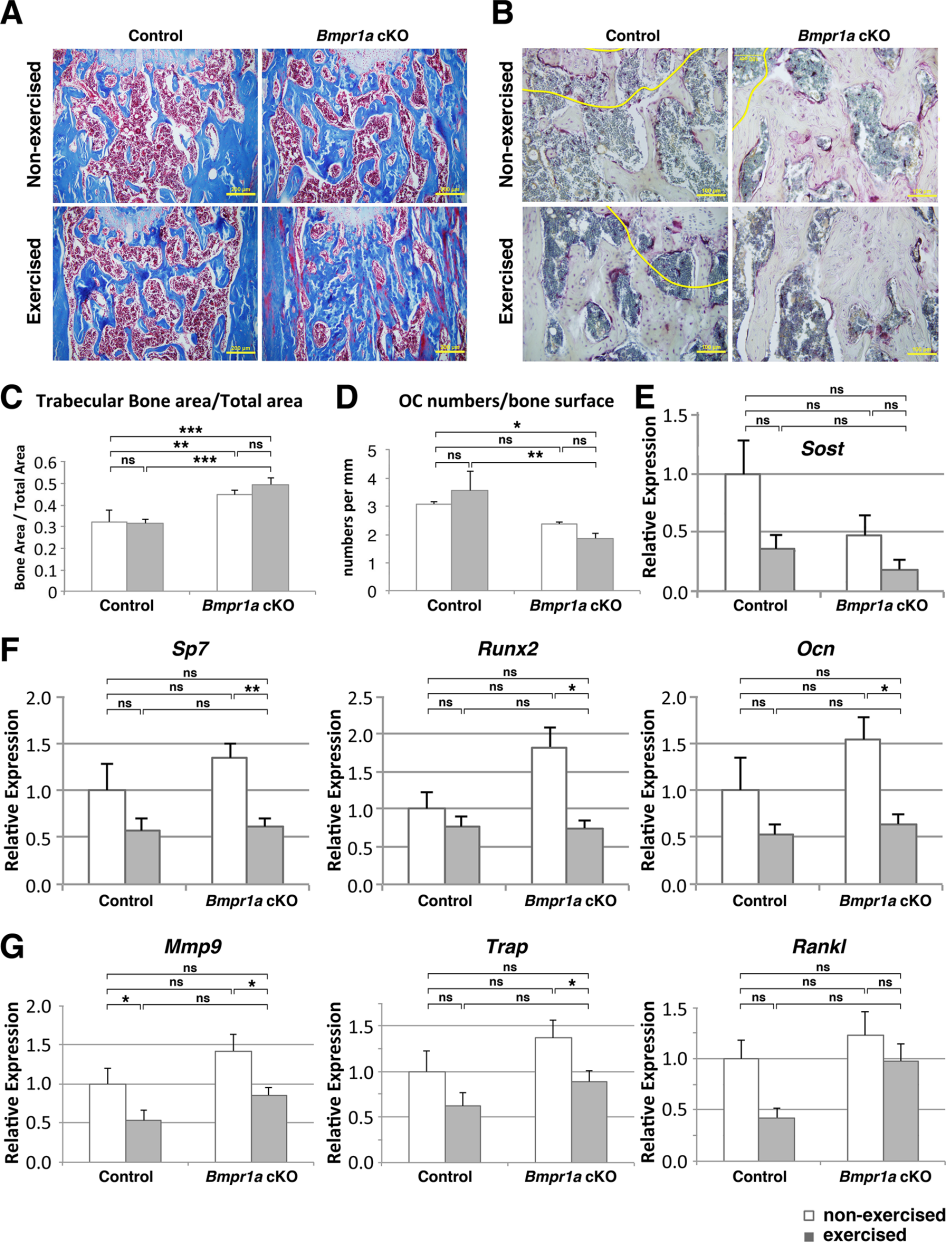
Mechanical loading increased trabecular bone area via decreased osteoclastogenesis in *Bmpr1a* cKO, whereas control bone was not affected. (A) Masson Trichrome staining showed collagen (blue) in bone and marrow cells (red) in the distal metaphyseal regions of femoral bone. An increase in collagen area was seen in exercised cKO bone. Bar = 200 μm. (B) TRAP staining on sections of femora. Osteoclast numbers, osteoclast surface, and total bone surface were measured using ImageJ. Nuclei were stained with hematoxylin (blue). Yellow lines represent 200 μm boundaries from the growth plate in the distal metaphysis. Bar = 100 μm. (C) Measurement of the trabecular bone area per total area of femoral metaphysis (BA/TA). (D) Osteoclast number per total bone surface (OC/BS) (E) PCR results showing levels of *Sost*. (F) Real-time PCR results showing levels of osteoblast markers (*Sp7*, *Runx2*, *Ocn*). (G) Real-time PCR results showing levels of osteoclast markers (*Trap*, *Mmp9*, *Rankl*). mRNA was harvested from tibial bone after 6 weeks of exercise or at the equivalent time in no-exercise mice. Mean±SEM, *, p<0.05, **, p<0.01, ***, p<0.001, ns, not significant.

The area of collagen matrix detected by Masson’s trichrome (blue in Fig 3A) in Nex cKO femora was increased compared with Nex controls (calculated as bone area/total area, BA/TA, p<0.05, Fig 3C). BA/TA showed a non-significant increase in the cKO femora with exercise, while in the control mice, BA/TA was not affected by exercise (Fig 3C). Exe cKO femora showed significantly larger BA/TA than Exe control (Fig 3C), similar to μCT results in the tibiae (Fig 1B). The Nex cKO bones showed a tendency to decrease in osteoclast numbers per total bone surface (OC/BS) compared with Nex control (Fig 3B and 3D). The cKO femora showed a tendency towards a decrease in OC/BS with exercise, whereas femora in control mice were not affected (Fig 3B and 3D). Exe cKO femora showed significant reduction in OC/BS when compared with Exe control bones (p<0.05, Fig 3D). Expression levels of *Sost* showed a tendency toward lower levels in cKO and also a tendency to further decrease after the exercise (Fig 3E). Osteoblast markers (*Sp7*, *Runx2*, *Ocn*) showed significant reductions with exercise only in cKO bones (p<0.001 for *Sp7*, p<0.05 for *Runx2* and *Ocn*, Fig 3F). *Alp* and *Col1a1* also showed significant reductions with exercise only in cKO bones (p<0.05, p<0.01, respectively), but no significant changes were observed in *Ibsp* (data not shown). Osteoclast markers (*Mmp9*, *Trap*) showed that *Mmp9* was downregulated by exercise in both cKO and control tibial bones (p<0.05), while *Trap* was downregulated by exercise in cKO and showed tendency to decrease in control tibia (Fig 3G). Gene expression was affected by exercise status, but no significant interaction with genotype was discovered by 2-way ANOVA. *Rankl* expression showed a tendency for downregulation by exercise in control bones (p = 0.069), but not in cKO bones (Fig 3G).

### Mechanical loading altered the diameters of collagen fibrils in an area-dependent manner

**Fig 4:**
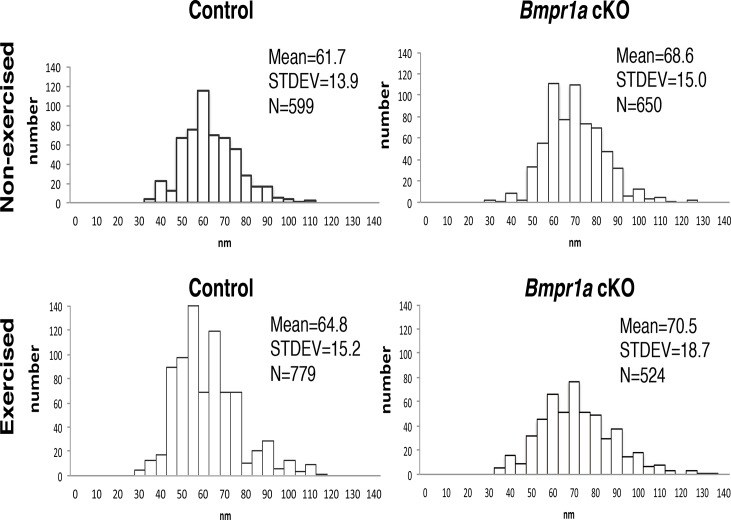
Mechanical loading altered diameters of collagen fibrils in area-dependent manner. Nonmineralized-bone collagen fibrils were randomly photographed at 46000-fold magnification using transmission electron microscopy (TEM), and diameters of collagen fibrils were measured in 4 groups (n≥3 samples, n≥500 collagen fibrils per group) Measurement of fibril diameters in cortical areas showed the same impact of exercise on the diameters in both groups.

We further examined collagen fibrils, since the diameter of the fibrils influences biomechanical properties of bone [30]. Collagen fibrils in the cortical compartment of Nex cKOs (mean 68.6 ± 15.0 nm) were significantly thicker than in Nex controls (61.7 ± 13.9 nm, p<0.0001), but the diameters increased significantly with exercise (64.8 ± 15.2 nm, p<0.0001 for control and 70.5 ± 18.7 nm, p<0.05 for cKO) (Fig 4).

### Exercise increased ductility and restored toughness of the cKO bones

**Fig 5:**
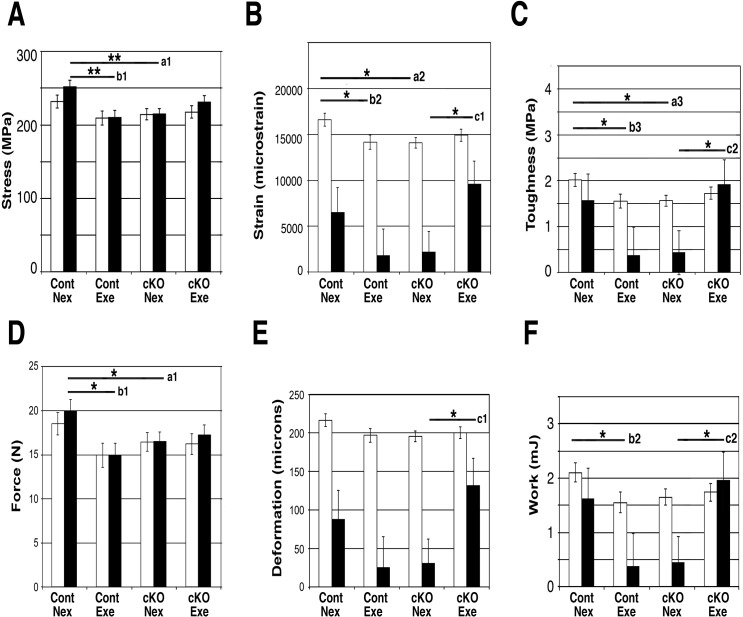
Mechanical loading restored toughness of cKO tibiae. Mechanical properties of the tibiae in each of the 4 genotype/exercise groups were measured by 4-point-bending. (A-C) Tissue-level properties, (A) Stress (yield stress (open bar), ultimate stress (solid bar)); cKO bones had significantly lower ultimate stress (a1) than control bones. The control mice had decreased ultimate stress with exercise (b1). (B) Strain (yield strain (open bar), post-yield strain (solid bar)); cKO bones had significantly lower yield strain (a2) than controls. The control mice had decreased yield strain with exercise (b2). Post-yield strain in cKO was increased by exercise (c1). (C) Toughness (pre-yield toughness (open bar), post-yield toughness (solid bar)); cKO bones had significantly lower pre-yield toughness (a3). Exercise significantly increased post-yield toughness (c2) in cKO bones, but pre-yield toughness in control bones declined with exercise (b3). (D-F) Whole bone properties; similar effects of genotype and exercise as at the tissue-level were found. (D) Force (yield force (open bar), ultimate force (solid bar)). (E) Deformation (yield deformation (open bar), post yield deformation (solid bar)), (F) Work (pre-yield work (open bar), post-yield work (solid bar)). Nex, non-exercised, Exe, exercised. Annotations next to bars; a, comparisons between Control Nex and cKO Nex. b, comparisons between control Nex and control Exe, c, comparisons between cKO Nex and cKO Exe. Mean±SEM; *, p<0.05, **, p<0.01.

Since exercise led to a marginally significant decrease in cortical porosity (Fig 2) and significant increases collagen fibril diameter in cKO mice (Fig 4), we hypothesized that the biomechanical properties of cKO and control bones may be differentially affected by exercise. Biomechanical properties were addressed at both the tissue (Fig 5A–5C) and whole bone levels (Fig 5D–5F). Bones from Nex cKO mice showed significantly decreased ultimate stress (Fig 5A a1), yield strain (Fig 5B a2), and pre-yield toughness (Fig 5C a3) in comparison with Nex controls. Bones from control mice that were exercised had decreased tissue-level properties compared with bones from Nex control mice, including significant decreases in ultimate stress (Fig 5A b1), yield strain (Fig 5B b2), and pre-yield toughness (Fig 5C b3). On the other hand, bones from Exe cKO mice had increased tissue-level properties compared with bones from Nex cKO mice, including significant increases in post-yield strain (Fig 5B c1) and post-yield toughness (Fig 5C c2). For all parameters shown, significant interactions between genotype and exercise were detected by 2-way ANOVA.

Bones from Nex cKO mice also showed decreased whole bone properties compared with bones from Nex control mice, including yield force (Fig 5D a1). Bones from Exe control mice showed decreased whole bone properties compared with bones from Nex controls, including yield force (Fig 5D b1), and pre-yield work (Fig 5F b2). On the other hand, exercise increased whole bone properties in the cKO mice compared to Nex cKO mice (post-yield deformation and post-yield work, Fig 5E c1 and 5F c2, respectively). Two-way ANOVA revealed significant interactions between genotype and exercise—exercise status interacts with genotype to modulate post-yield deformation and post-yield work.

## Discussion

In this study, we applied mechanical loading through running on a treadmill to adult *Bmpr1a* cKO mice for 6 weeks to elucidate the synergistic impact of loss of BMP signaling and mechanical loading on bone mass and biomechanical properties. In the Nex cKO animals, the bones developed increased bone volume and mineral density in tibial trabecular bone. In the cortical compartment, no genotypic changes in bone volume and mineral density were found, but cortical porosity increased, consistent with previous reports [13–15]. In the Exe mice, *Bmpr1a* cKO bones showed further increased trabecular bone volume (tibia) and mineral bone density (tibia, femur) compared with bones from Nex cKO mice, while control bones showed no changes in these parameters with exercise (Fig 1). Expression levels of several osteoblast marker genes were not increased by exercise, rather they were decreased (Fig 3). Expression of some of the osteoclast marker genes was also decreased by exercise (Fig 3). Although marginally, expression levels of *Sost* were reduced when Exe cKO bones were compared with Nex control bones (Fig 3). Exe cKO tibia showed lower osteoclast numbers per bone surface when compared with Exe control tibia (Fig 3). Since mRNA was extracted from whole tibiae, we need to be mindful that some results might be affected by the presence of marrow cells in the samples. These results suggest that exercise and loss of BMPR1A signaling in osteoblasts together decrease osteoblast functions that are necessary to support osteoclastogenesis, leading to a further increase in trabecular bone volume and mineralization (Figs 1 and 3).

The increased cortical porosity found in bones from Nex cKO mice show a trend toward to normal levels with exercise (Fig 2). In control mice, exercise did not change porosity, while the mutants had significantly increased porosity relative to controls (p = 0.025) and showed a marginally significant decrease in porosity with exercise (p = 0.061), and a tread to adapt to normal levels. Since no other changes were found between the Nex and Exe cKO groups in the cortical compartment, these results suggest that osteoblasts without functional BMP signaling are more sensitive to the pore-preventing effects of exercise. We do not know whether the decrease in porosity in the exercised mice is due to the formation of pores being prevented or pores being filled during exercise. This decrease of porosity may have led to the increase in biomechanical properties in cKO tibiae (Fig 5).

The Nex cKO bones have decreased whole bone and tissue-level biomechanical properties (ultimate stress, yield strain, and pre-yield toughness) in comparison with Nex control bones (Fig 5). Although the exercise regime used in this study resulted in decreased biomechanical properties in control bones, the Exe cKO group displayed an increase in ductility, namely, restoring toughness and work at the tissue and whole bone levels, respectively. Mechanical properties using the 4-point-bending test are determined on the cortical compartment and thus we speculate that the marginally significant reduction in cortical porosity, despite no changes in cortical bone volume in the Exe cKO bones contributed to the increased mechanical properties. Increased porosity of human cortical bone, measured as average pore diameter, is related to decreasing material properties [16,31,32]. There is evidence that age- or disease-related increases in cortical porosity also negatively affect mechanical properties and increase risk of fracture [33,34].

Previously, we reported that 3 weeks of exercise on a treadmill led to increased structural post-yield deformation, but decreased yield deformation [22,23]. In this study, we used the same speed and incline, and mice were of the same genetic background. However, we exercised the mice for 6 weeks instead of 3 weeks and started exercise at 11 weeks of age vs. 8 weeks. The differences in age at which exercise started and exercise duration may have led to the decrease in ductility in the control mice. An alternative explanation for the different outcomes in control bones from the previous reports [22,23] is that all of the mice used in this study received tamoxifen (TM) to induce a conditional deletion of *Bmpr1a*. TM can prevent bone loss because it acts as an agonist for the estrogen receptor [35,36], however, its effect on males, especially in combination with exercise, is not fully understood. Nonetheless, our results clearly demonstrate that osteoblasts deficient in BMP signaling through BMPR1A differentially respond to exercise to improve biomechanical properties of bones compared to control exercised groups.

Besides in the decrease in cortical porosity in Exe cKO bones, several other changes in the microstructure of these bones may also influence the biomechanical properties. Since the changes in tissue-level properties paralleled the changes in whole bone properties, but no differences in cortical cross-sectional geometry were detected, we can infer that the whole bone changes were driven by the tissue-level changes. Bone mineral provides mechanical rigidity and load-bearing strength to bone, whereas the organic matrix provides toughness [37]. Crosslinking within the collagen matrix also plays an important role in determining strength and toughness [38]. We found that the diameter of the collagen fibrils is affected differently by the loss of BMPR1A signaling and alterations in mechanical loading. In the cortical compartment, the diameter of the collagen fibrils was larger in the cKO, and mechanical loading further increased the diameter (Fig 4). These results suggest that post-translational modifications of collagen fibrils, including cross-linking, may be affected by mechanical loading, and may induce this compartmental-specific difference. It is also possible that slower turnover of bone matrix due to reduced osteoclastogenesis in Exe cKO bones may contribute to the larger diameter of collagen fibrils. The increased diameters in exercised cKO bones may contribute to the increase in ductility (higher post-yield deformation in exercised cKO bones, Fig 5E, c1).

There are several potential explanations for the discrepancy between changes in fibril diameter and changes in post-yield deformation between control and cKO bones. Although the increase in average collagen fibril diameter with exercise is comparable between the two genotypes (5.0% for control, 2.8% for cKO), the distribution of diameters is different between control and cKO bones (Fig 4), which may differentially affect mechanical properties. Another explanation is based on a difference in cause and effect between the 2 genotypes. In control mice, exercise may have led to the increase in fibril diameter, which in turn led to a decrease in post-yield deformation. However, in cKO mice, the reduced post-yield deformation may have triggered a need to adapt in response to this compromise in function and, as a result, exercise resulted in a large (6-fold) increase in post-yield deformation (larger than the decrease in control mice with exercise) that is driven by compositional parameters other than fibril diameter.

Our study showed, for the first time to the best of our knowledge, that mice with osteoblast-specific deletion of BMPR1A signaling respond to mechanical loading differently from control mice. Reduction in mechanical stress leads to significant bone loss as evidenced by patients who are bedridden for long times and in astronauts during space flight [4,39–41]. Our results suggest that suppression of BMPR1A signaling in osteoblasts may enhance load-stimulated bone adaptation. Mechanical loading in combination with *Bmpr1a* deficiency showed improved bone quality. With this type of forced exercise, it is important to rule out the possibility of a stress response. No differences in hormone levels, geometric or mechanical properties have been found between non-exercise mice that are cage-bound and non-exercise mice that are placed on the treadmill and subjected to the same stimuli as the exercise group except for the exercise itself [22–24,29]. These results suggest that the effects of this exercise regime are the result of mechanical loading and not a stress response. Mechano-sensing is likely not exclusively controlled by BMPR1A, and BMP signaling is likely linked with other signaling pathways to control mechanotransduction. Since we selected treadmill to apply mechanoloading to the mice, it will be an important future study to evaluate a possible impact of alterations in general metabolism. It will also be an interesting direction to study potential involvement of other signaling pathway and how their synergistic interaction with BMP signaling together affecting mechano-sensing machineries.

## Supporting Information

S1 Fig Experimental design for long-term (6 weeks) exercise. Homozygous male mice for a floxed allele of *Bmpr1a* (*Bmpr1a* fx/fx), aged 9–10 weeks (17 *Col1-CreERTM* positive (cKO) and 14 *Col1-CreERTM* negative (control) mice) were used. All mice were injected with tamoxifen intraperitonially twice a week at the 9^th^ and 10^th^ week and once a week at 11^th^ to 16^th^ week. The exercised groups of mice ran on a motor-driven treadmill 5 days/week for 6 weeks from 11 to 16-weeks of age. Each exercise session lasted 30 minutes and the average speed was 12 ±1m/min at a 5°incline.
(TIF)

S2 Fig Body weight increased with exercise in both control and cKO mice. The rate of body weight increase in exercised cKO was 9.2% higher than non-exercised cKO, whereas exercised control mice were only 2.3% higher than non-exercised control mice. Mean±SEM, *, p<0.05, **, p<0.01, ***, p<0.001.
(TIF)

S3 Fig Geometry, BMD and biomechanical properties of tibial cortical compartments. No difference in geometry or BMD at the standard site of male tibial cortical bone. (A) Geometry, (B) Bone mineral density, (C) Cortical thickness. Mechanical properties of the tibia in 4 groups were measured by a 4-point-bending test. Tissue properties, (D) Modulus; whole bone properties, (E) Stiffness. No changes were detected, Comparisons were made between control and cKO, and no-exercised (Nex) and exercised (Exe) groups.
(TIF)
