# Supplementary figures and images for: Mechanical Loading Synergistically Increases Trabecular Bone Volume and Improves Mechanical Properties in the Mouse when BMP Signaling Is Specifically Ablated in Osteoblasts
Source: PLoS One. 2015 Oct 21;10(10):e0141345. doi: 10.1371/journal.pone.0141345 (PMC4619208; doi:10.1371/journal.pone.0141345)

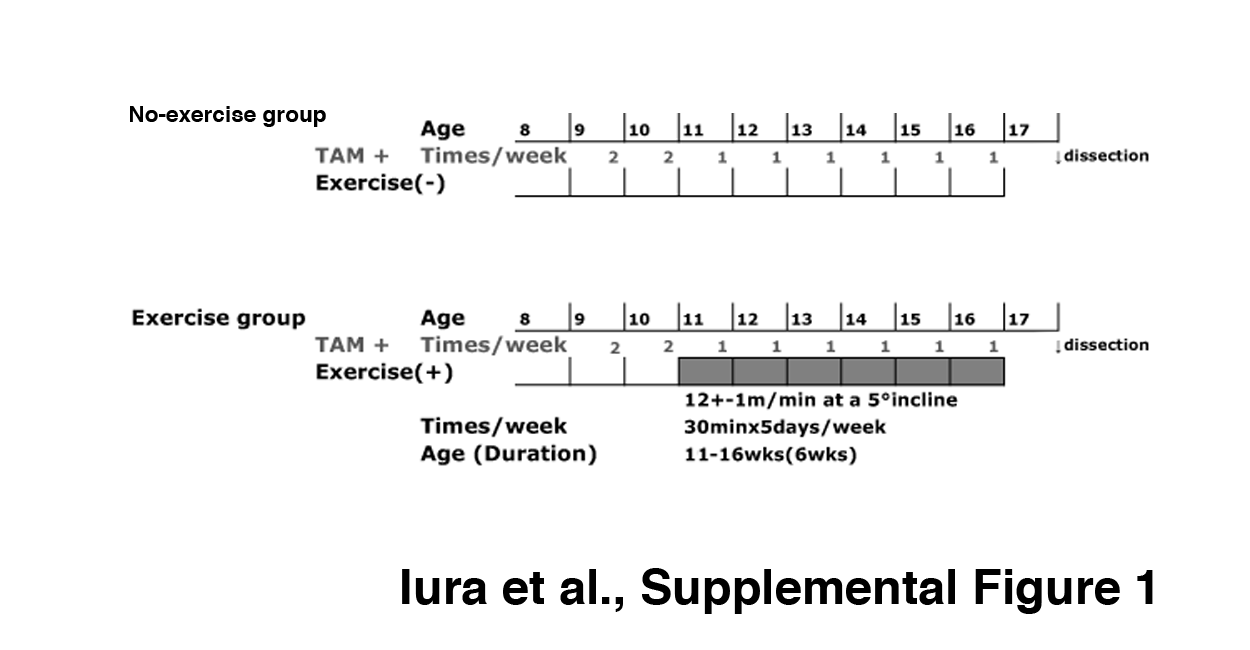

Supplement: S1 Fig — Homozygous male mice for a floxed allele of Bmpr1a (Bmpr1a fx/fx), aged 9–10 weeks (17 Col1-CreERTM positive (cKO) and 14 Col1-CreERTM negative (control) mice) were used. All mice were injected with tamoxifen intraperitonially twice a week at the 9th and 10th week and once a week at 11th to 16th week. The exercised groups of mice ran on a motor-driven treadmill 5 days/week for 6 weeks from 11 to 16-weeks of age. Each exercise session lasted 30 minutes and the average speed was 12 ±1m/min at a 5°incline. (TIF) [file pone.0141345.s001.tif]

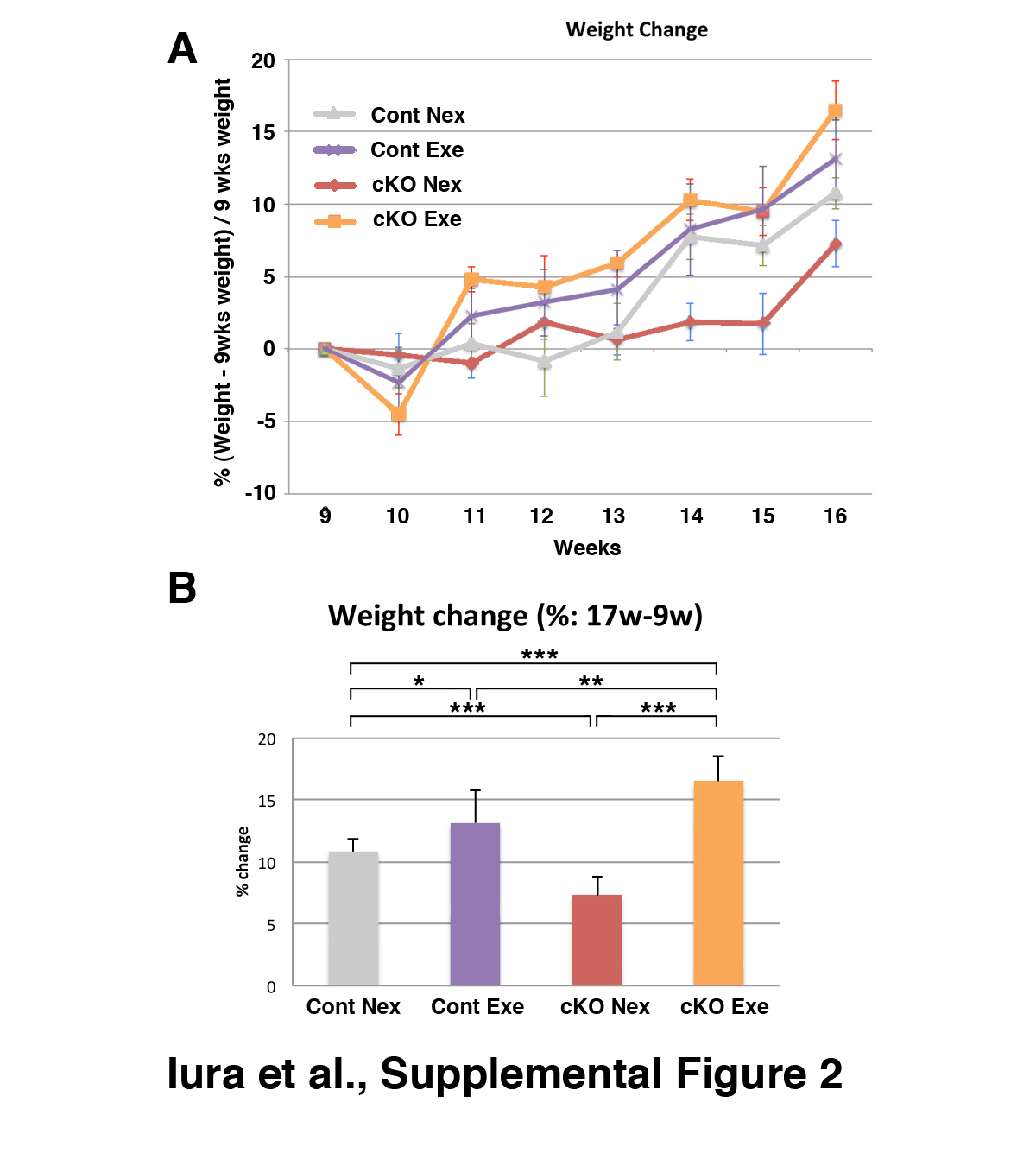

Supplement: S2 Fig — The rate of body weight increase in exercised cKO was 9.2% higher than non-exercised cKO, whereas exercised control mice were only 2.3% higher than non-exercised control mice. Mean±SEM, *, p<0.05, **, p<0.01, ***, p<0.001. (TIF) [file pone.0141345.s002.tif]

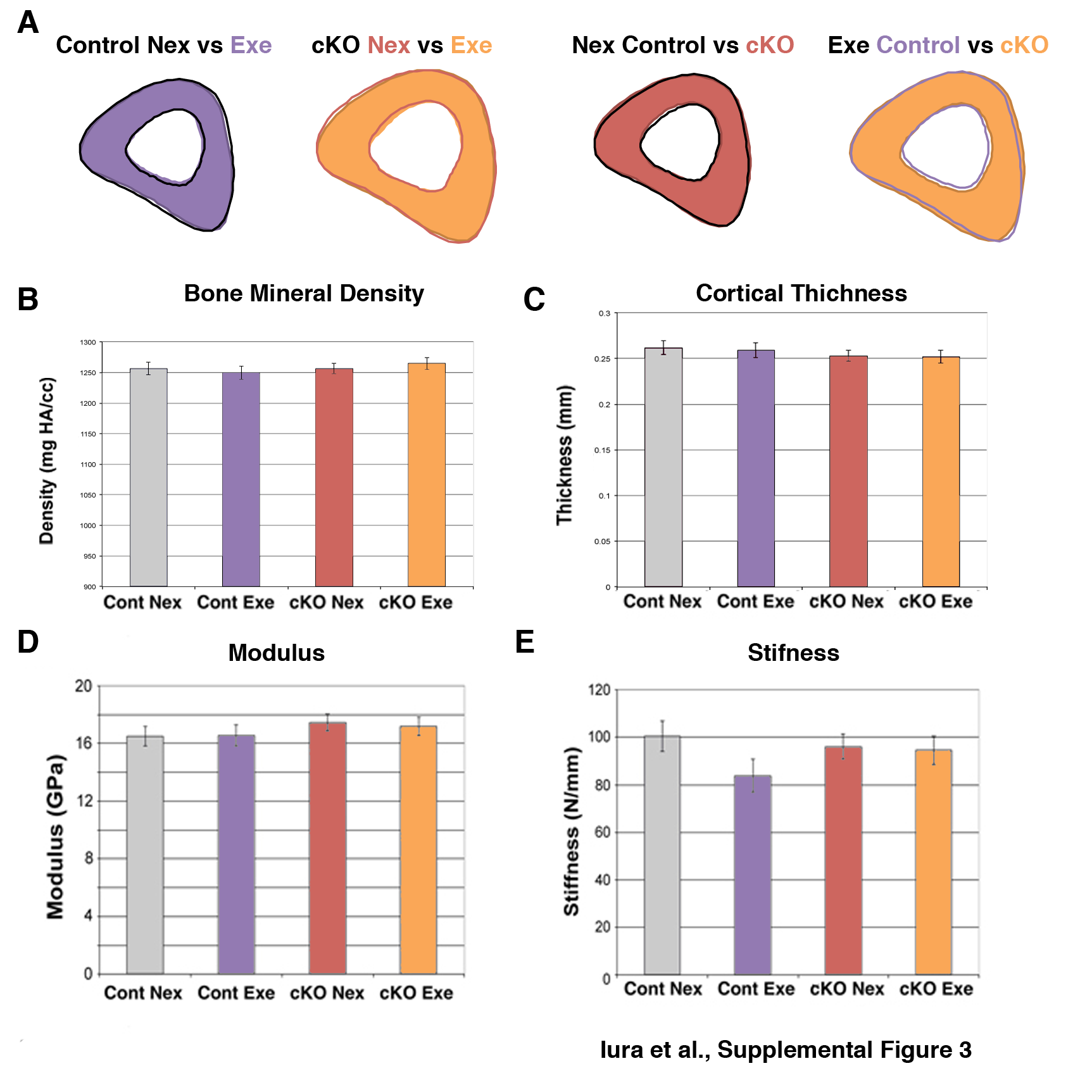

Supplement: S3 Fig — No difference in geometry or BMD at the standard site of male tibial cortical bone. (A) Geometry, (B) Bone mineral density, (C) Cortical thickness. Mechanical properties of the tibia in 4 groups were measured by a 4-point-bending test. Tissue properties, (D) Modulus; whole bone properties, (E) Stiffness. No changes were detected, Comparisons were made between control and cKO, and no-exercised (Nex) and exercised (Exe) groups. (TIF) [file pone.0141345.s003.tif]
